# A Case of Multiple Myeloma Associated with Extramedullary Plasmacytoma of the Gallbladder Manifesting as Acute Cholecystitis

**DOI:** 10.7759/cureus.2688

**Published:** 2018-05-25

**Authors:** Omar Abughanimeh, Ayman Qasrawi, Mohannad Abu Omar, Waled Bahaj, Mouhanna Abu Ghanimeh

**Affiliations:** 1 Department of Internal Medicine, University of Missouri Kansas City School of Medicine, Kansas City, USA; 2 Department of Internal Medicine-Gastroenterology, Henry Ford Health System, DETROIT, USA

**Keywords:** multiple myeloma, acute cholecystitis, plasmacytoma, gall bladder

## Abstract

Multiple myeloma (MM) is a common hematological malignancy that represents 1% of all cancers. MM is distinguished from other plasma cell disorders by prominent bone marrow involvement and systemic organ damage. Extramedullary plasmacytomas of the gall bladder (GB) or biliary ducts, whether solitary or in association with MM, are very rare. We report a case of a 66-year-old female with a history of refractory MM who presented with right upper quadrant abdominal pain. Her laboratory evaluation revealed an abnormal liver panel and lactic acidosis. Abdominal ultrasonography was consistent with acute cholecystitis with no evidence of biliary obstruction or abnormal liver parenchyma. An open cholecystectomy with liver biopsy was performed. The histological evaluation revealed involvement of the GB submucosa and serosa, as well as the liver parenchyma by abnormal plasma cells with lambda light chain restriction. Congo red stain for the GB sample was positive. The patient declined further treatment for MM and was discharged home with comfort measures.

## Introduction

Multiple myeloma (MM) is a common hematological malignancy characterized by the malignant proliferation of clonal plasma cells with the overproduction of monoclonal proteins [1-3]. MM is considered the most common primary tumor originating from bone [1]. The annual incidence of MM is approximately 4 to 5 per 100,000 people [4]. Solitary plasmacytomas are localized plasma cell tumors that arise in the bone (solitary plasmacytoma of the bone) or outside the bone in the soft tissues (solitary extramedullary plasmacytoma) [1-2,5]. Solitary plasmacytomas typically lack the systemic manifestations of MM. However, MM can be complicated by secondary plasmacytomas, which are usually associated with a more aggressive course, shorter progression-free survival, and worse prognosis [1,4,6-7]. The involvement of the gall bladder (GB) and biliary ducts by plasma cell neoplasms, whether solitary plasmacytomas or MM with secondary plasmacytomas, is rare [7-14]. The clinical and radiological manifestations of GB involvement can be confused with many benign and malignant diseases. Thus, GB involvement of MM is rarely considered in the differential diagnosis of GB diseases, and the majority of the cases are diagnosed post-operatively or post-mortem [8].

## Case presentation

A 66-year-old African American female with a past medical history of refractory immunoglobulin G (IgG) lambda MM, essential hypertension, and chronic kidney disease presented to the emergency department with five days of right upper quadrant pain.

Her MM was diagnosed one year prior when she presented with altered mental status, uremia, hypercalcemia, hypoalbuminemia, and paraproteinemia. A skeletal survey at that time revealed multiple thoracic spinal lytic lesions and an eroding soft tissue mass at the level of T10. Further evaluation revealed a very high IgG level, elevated M protein band, and a kappa/lambda ratio <0.01 (normal 0.26-1.65). A biopsy from the soft tissue mass revealed a plasmacytoma. Radiation therapy was initiated for 10 days. She received three cycles of bortezomib and dexamethasone followed by two cycles of bortezomib, dexamethasone, and lenalidomide. Her disease progressed, and a subsequent bone marrow biopsy revealed hypercellular bone marrow with 70% atypical plasma cells. The patient subsequently received seven cycles of carfilzomib, lenalidomide, and dexamethasone. She was not a candidate for bone marrow transplantation given the high plasma cell burden.

On her current presentation, the pain was sudden in onset, intermittent, worse with eating, and without radiation. The pain was associated with nausea and anorexia, but she was without any change in bowel habits. She denied any previous similar episodes. Upon physical exam, the patient was in distress but remained alert and oriented. Her vital signs were all stable. She exhibited right upper quadrant abdominal tenderness without rebound or guarding. Her initial labs are presented in Table 1. The patient was admitted to the hospital for further evaluation of her abnormal labs and supportive treatment.

**Table 1:** Patient’s initial laboratory workup

Table 1: Patient’s initial laboratory workup	
Alanine aminotransferase	400 U/L (normal 14-54 U/L)
Aspartate aminotransferase	222 U/L (normal 15-41 U/L)
Total bilirubin	5.5 U/L (normal 0.3-1.4 mg/dl)
Direct bilirubin	2.7 U/L
Alkaline phosphatase	110 U/L (normal 32-91 U/L)
Albumin	2.1 g/dL (normal 3.5-4.8 g/dl)
Hemoglobin	8.1 (normal 12-16 g/dl)
White blood cell count	6,700/cmm (normal 4,300-10,800/cmm)
Creatinine	2.02 mg/dL (normal 0.9-1.3 mg/dl)
Blood urea nitrogen	40 mg/dL (normal 8-20 mg/dl)
Total protein	11.4 g/dL (normal 6.1-7.9 g/dl)
Lactic acid	5.3 mmol/L (normal 0.5-2.2 mmol/L)

**Figure 1:**
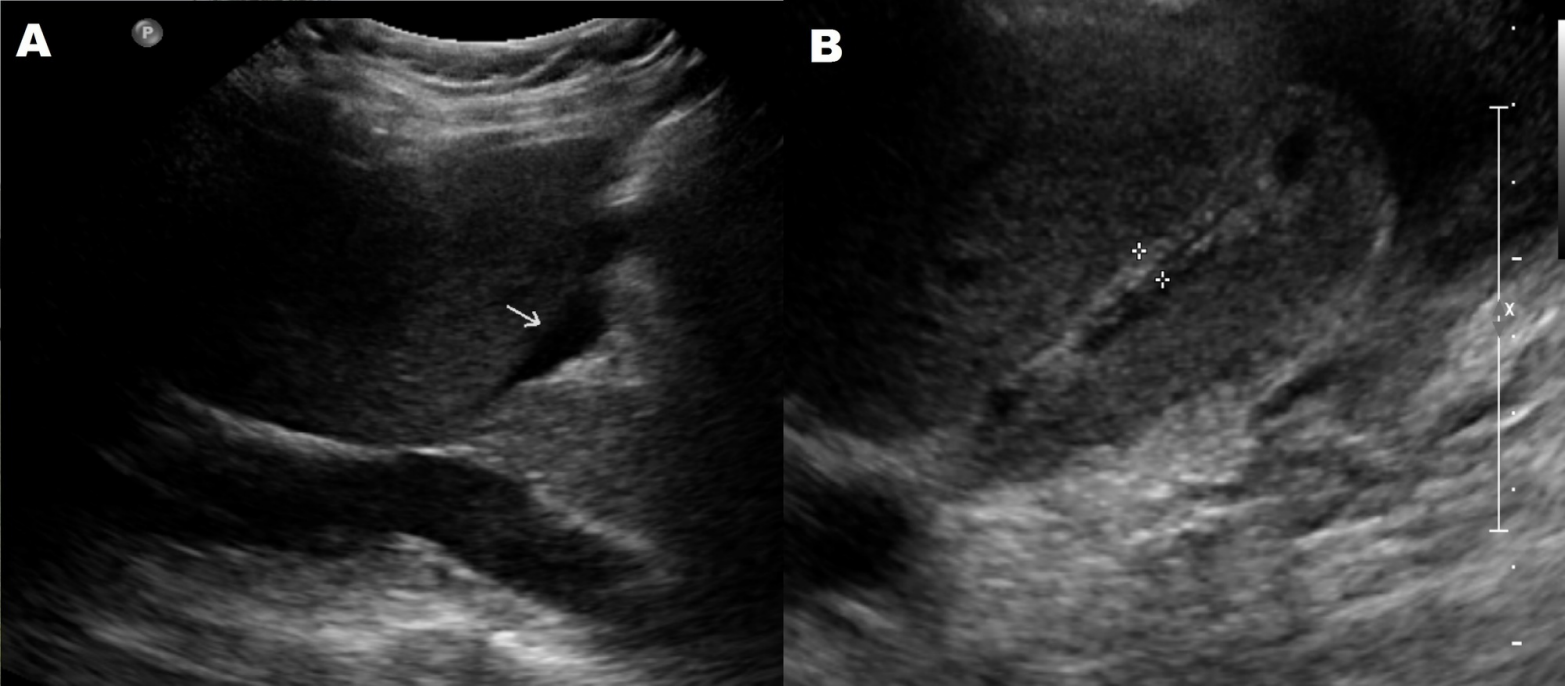
An abdominal ultrasound showing a distended gall bladder (GB) with pericholecystic fluid (A) and a thickened GB wall (B)

The patient was started on intravenous hydration and was made nil per os. An abdominal ultrasound revealed a distended GB with sludge (Figure 1). The GB wall was thickened up to 9.5 mm, and the sonographic Murphy sign was positive. The common bile duct and common hepatic duct measured 6.3 mm and 3 mm, respectively. The liver measured 18.3 cm and exhibited normal echogenicity. There were no intraparenchymal masses or fluid collections. The portal and hepatic veins were patent.

The patient was diagnosed with acute cholecystitis. Intravenous piperacillin-tazobactam 3.37 g every eight hours was initiated, and the patient was referred for open cholecystectomy given her overall condition and lactic acidosis. Intra-operatively, the GB was thickened and firm but not overly distended or perforated. The GB was dissected from the liver edge, and a liver biopsy was performed successfully.

**Figure 2:**
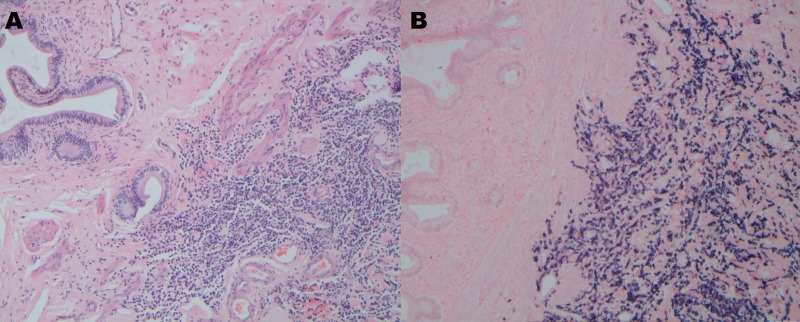
Hematoxylin and eosin stain (A) and lambda light restriction (B) of the gall bladder (GB) showing chronic cholecystitis with involvement of the GB submucosa and serosa by abnormal plasma cells with lambda light chain restriction

The pathology report from the cholecystectomy revealed chronic cholecystitis with involvement of the GB submucosa and serosa by abnormal plasma cells with lambda light chain restriction (Figure 2). Subsequently, Congo red stain of the GB sections revealed apple-green birefringence throughout the submucosal areas consistent with amyloid deposits. The liver biopsy exhibited abnormal plasma cells in periportal locations with lambda light chain restriction as well; Congo red stain was not done on the liver sample.

The patient declined any further treatment for MM and decided to proceed with hospice care. She was discharged home with comfort measures.

## Discussion

Plasma cell neoplasms are a group of very diverse diseases characterized by neoplastic proliferation of clonal plasma cells and the production of monoclonal immunoglobulin or free light chains. These neoplasms can present as MM with or without extramedullary extension, solitary plasmacytoma of the bone, solitary extramedullary plasmacytoma, or plasma cell leukemia [1-2,5].

MM is a malignant plasma cell neoplasm that represents approximately 10% of all hematological malignancies and 1% of all cancers [1-3]. MM is a disease of the elderly, with a median age at diagnosis between 66 and 69 years [15]. The clinical symptoms and signs of MM are a direct result of the uncontrolled proliferation of clonal plasma cells and the overproduction of monoclonal proteins and light chains [1]. A single-institution large retrospective analysis included 1027 MM patients and reported that anemia (73%) and bone pain (58%) are the most common presenting symptoms [16].

The diagnosis of MM is very important and should be distinguished from solitary plasmacytomas. Solitary plasmacytomas are considered distinct clinical diseases from MM with different treatments and prognoses [1,4,6-7]. The international myeloma working group [17] established the criteria for MM. These criteria indicate that prominent bone marrow involvement (clonal bone marrow plasma cells ≥10%) and organ damage/dysfunction (hypercalcemia, renal insufficiency, anemia, or bone lesions) are crucial in the diagnosis of MM [17].

Extramedullary plasmacytomas have been increasingly detected with MM [6]. This increase may be attributed to the improvement in the survival rates of MM patients as well as to the advanced evaluation techniques. In most cases, extramedullary plasmacytomas are the result of direct extension in the bone, but hematogenous spread involving distant organs may occur [2].

Extramedullary plasmacytomas are observed in approximately 7%-18% of patients with newly diagnosed MM [18]. More patients develop extramedullary plasmacytomas in the later stages of the disease. As per Oshima et al., post-mortem evaluation of MM patients revealed that extramedullary plasmacytomas or extraosseous spread may occur in up to two-thirds of patients [19]. Reticuloendothelial organs including the spleen, liver, and lymph nodes are the most common organs involved according to the post-mortem evaluation [19]. In contrast, 80% of solitary extramedullary plasmacytomas are located in the head and neck region, primarily in the upper aerodigestive tract [11].

GB and biliary involvement by extramedullary plasmacytoma, whether solitary or as a secondary infiltration associated with MM, is rare and has only been described in case reports [7-14]. Such involvement may manifest similarly to primary cholangiocarcinoma, primary GB cancer or other common benign inflammatory, infectious, or obstructive diseases of the GB and biliary ducts [7-14]. Therefore, these extramedullary plasmacytomas are not typically considered in the differential diagnosis of biliary diseases, and the majority of cases are detected post-operatively or post-mortem [8].

Clinically, extramedullary plasmacytomas of the GB or biliary ducts can be asymptomatic or manifest as obstructive jaundice, vague abdominal pain, or, rarely, as acute cholecystitis with constant abdominal pain, tenderness to palpation, and positive Murphy sign [7-14]. Radiologically, a thickened GB wall, dilated biliary ducts, or enlarged lymph nodes can be detected [7-14].

Per our literature search, eight cases of extramedullary plasmacytomas of the GB or biliary ducts were found [7-14]. Four cases were associated with MM, [9-10,12,14] and the other four were for solitary [7-8,11,13]. Table 2 summarizes these cases. To the best of our knowledge, our case represents the first report in the English literature of MM with secondary extramedullary plasmacytoma manifesting as acute cholecystitis. One case of solitary extramedullary plasmacytoma manifesting similarly was reported in Germany. In conclusion, although secondary malignant infiltration of the GB and biliary system is uncommon, it should be considered in the differential diagnosis of GB diseases, especially in patients with a history of malignancy or concerning symptoms.

**Table 2:** Reported cases of MM/plasmacytoma with GB/biliary involvement MM: multiple myeloma; GB: gall bladder.

Table 2: Reported cases of MM/plasmacytoma with GB/biliary involvement			
Case	Plasma cell disorder	Age (years old) and Gender	Presenting gastrointestinal symptoms
Kondo et al.1995 [7]	Solitary extramedullary plasmacytoma, No MM	53, male	Painless obstructive jaundice
Hwang et al. 2010 [8]	Solitary extramedullary plasmacytoma, No MM	63, female	Painless obstructive jaundice
Majerović et al.2012 [11]	Solitary extramedullary plasmacytoma, No MM	69, male	Right upper quadrant pain (diagnosed 5 months after laparoscopic cholecystectomy in the GB fossa)
Schuster et al. 2007 [13] *German	Solitary extramedullary plasmacytoma, No MM	66, male	Right upper quadrant pain, acute cholecystitis
Romain et al.2015 [9]	MM with secondary extramedullary plasmacytoma	53, female	Incidental imaging finding which was done as part of evaluation of abnormal liver panel and graft versus host disease
Heckmann et al. 2008 [10]	MM with secondary extramedullary plasmacytoma	70, male	Right upper quadrant pain with no evidence of acute cholecystitis
Fukatsu et al. 2013 [12]	MM with secondary extramedullary plasmacytoma	80, male	Painless obstructive jaundice
Abt et al.1969 [14]	MM with secondary extramedullary plasmacytoma	53, female	Incidental finding after working up an anemia in patient admitted for elective cholecystectomy

Our case was presented in the World Congress of Gastroenterology/American College of Gastroenterology annual meeting 2017. (Abu Ghanimeh M, Abughanimeh O, Abu Omar M, Qasrawi A, Kaddourah O, Saettele T. A Case of Multiple Myeloma Associated With Extramedullary Plasmacytoma of the Gallbladder Manifesting as Acute Cholecystitis (Abstract). Am J Gastroenterol 2017; 112 (S1):S752–S753; DOI:10.1038/ajg.2017.311. PMID: 28981027.)

https://eventscribe.com/2017/wcogacg2017/ajaxcalls/PosterInfo.asp efp=S1lVTUxLQVozODMy&PosterID=115499&rnd=0.8310647)

## Conclusions

MM is a malignant plasma cell neoplasm. Solitary plasmacytomas are considered distinct clinical diseases from MM with different treatments and prognoses. However, MM can be complicated by secondary plasmacytomas, which are usually associated with a more aggressive course. GB and biliary involvement by extramedullary plasmacytoma, whether solitary or as a secondary infiltration associated with MM, is rare and has only been described in case reports. Secondary malignant infiltration of the GB and biliary system should be considered in the differential diagnosis of GB diseases, especially in patients with a history of malignancy or concerning symptoms.
